# Leisure-time sport and overuse injuries of extremities in children age 6–13, a 2.5 years prospective cohort study: the CHAMPS-study DK

**DOI:** 10.1136/bmjopen-2016-012606

**Published:** 2017-01-13

**Authors:** Charlène Chéron, Charlotte Leboeuf-Yde, Christine Le Scanff, Eva Jespersen, Christina Trifonov Rexen, Claudia Franz, Niels Wedderkopp

**Affiliations:** 1 Institut Franco-Européen de Chiropraxie, 72 Chemin de la Flambère, Toulouse, France; 2 CIAMS, Univ. Paris-Sud, Université Paris-Saclay, Orsay Cedex, France; 3 Spine Center of Southern Denmark, Institute of Regional Health Services Research, University of Southern Denmark, Hospital Lillebaelt, Middelfart, Denmark; 4 Department of Rehabilitation, Odense University Hospital, Odense, Denmark; 5 Institute of Sports Science and Clinical Biomechanics, Center for Research in Childhood Health, University of Southern Denmark, Odense, Denmark; 6 Orthopaedic Department, Sport Medicine Clinic, Hospital of Lillebaelt—Middelfart, Institute of Regional Health Services Research, Middelfart, Denmark

**Keywords:** EPIDEMIOLOGY, children, adolescent, overuse injuries, extremities, sport type

## Abstract

**Objectives:**

It is not known which sports are most likely to cause overuse injuries of the extremities in children. In this study, we report on the incidence of overuse injuries of the upper and lower extremities in children who participate in various leisure-time sports and relate this to the frequency of sport sessions.

**Design:**

Natural experiment including a prospective cohort study.

**Setting:**

10 state schools in 1 Danish municipality: Svendborg.

**Participants:**

1270 children aged 6–13 years participating in the Childhood Health, Activity, and Motor Performance School Study Denmark.

**Outcomes measures:**

Over 2.5 years, parents answered weekly SMS-track messages (a) on type and frequency of leisure-time sports undertaken by their child, and (b) reporting if their child had experienced any musculoskeletal pain. Children with reported pain were examined by a clinician and diagnosed as having an overuse injury of an extremity or not. The incidence of diagnosed overuse injury was calculated for each of the 9 most common sports in relation to 5-week periods. Incidence by frequency of sessions was calculated, and multivariable analysis was performed taking into account age, sex and frequency of physical education classes at school.

**Results:**

Incidence of overuse injuries of the lower extremity ranged from 0.2 to 3.3 for the 9 sports, but was near 0 for overuse injuries of the upper extremities. There was no obvious dose–response. The multivariate analysis showed soccer and handball to be the sports most likely to result in an overuse injury.

**Conclusions:**

Among a general population of schoolchildren, overuse injuries of the lower extremities were not common and overuse injuries of the upper extremities were rare. Organised leisure-time sport, as practised in Denmark, can be considered a safe activity for children.

**Strengths and limitations of this study**

- This is the first study to determine the incidence of overuse injuries of extremities in different types of sports in the general population.
- Another strength is the large number of participants and that data were collected weekly over 2.5 years.
- In addition, the diagnosis of overuse injury was made in a physical examination following a predetermined procedure.
- The mean weekly incidence rate was reported based on the exposure during the 5 weeks preceding the occurrence of the injury in order to be in line with the concept of overuse injury: the repetitive activity takes place during a given time. However, this strategy makes comparison with other studies difficult, as the exact number of hours per person fails to appear.
- However, the data did not allow for analysis of injuries when children participated in more than one sport at a given time.

## Introduction

Injuries from sport and physical training are the major cause of school-related^1^ and leisure-time injuries in children.^2^ The consequences may be absence from school and sport, and need for medical attention.^3^ Injuries incurred in childhood may also lay the foundation for future problems extending into adulthood, and perhaps even permanent disability.

Sports medicine deals with two main types of injuries, commonly defined according to the presumed cause: trauma or overuse. Traumatic injury results from a single, specific and identifiable event, while overuse injury occurs after repeated microtrauma without a single, identifiable event responsible for the injury.^4^

Several sports-specific studies with children and adolescents suggest that traumatic injuries of the extremities are more common than overuse injuries, at least when reported in relation to time-loss.^5–9^ In contrast, overuse injuries of the extremities (OIE) were more common than traumatic injuries in a large Danish school population.^10^ However, this study defined ‘injury’ according to a diagnosis after a clinical examination. Thus, after reporting pain, the child was seen by a clinician and if the child still had the reported symptoms, he or she was examined and diagnosed by a clinician. A dose–response was noted for traumatic and overuse injuries of the lower extremities, with a 20% increased risk for each exposure to leisure-time sport.^10^ No distinction was made between different types of sport, however.^11^

A systematic review of the literature indicated that overuse injuries in young people were more common in the lower extremities than in the upper extremities.^12^ However, although each of the reviewed studies dealt with only one type of sport, it was not possible to compare sport-specific injury incidence due to different methods and unreported information on site of injury and incidence.

The objectives of the present study were:

1. To confirm if overuse injuries in children are more common in the lower extremities than in the upper extremities for specific types of sport;
2. To estimate the incidence of diagnosed overuse injuries of the upper and lower extremities in relation to exposure (dose–response) for specific types of sport, with or without ensuing time-loss;
3. To investigate whether some types of sport are more likely than others to be associated with overuse injuries of the lower extremities, after controlling for relevant variables.

It is to be noted that the diagnosis of injuries was made because there was an initial report on pain and not because the child initiated a medical consultation.

## Method

### Setting

Data were extracted from a large prospective controlled study of children attending Danish public schools that was part of a natural experiment on additional physical education in school (the Childhood Health, Activity, and Motor Performance School Study Denmark, CHAMPS-study DK). The purpose of the CHAMPS project was to evaluate the effects of increased physical education on childhood health and well-being. It has been described in more detail elsewhere,^13^ and a number of studies have been published.^10^ ^11^ ^14^

The 19 schools in the municipality of Svendborg were invited to participate in the CHAMPS-study DK. Of these, six became sport schools and had six physical education lessons per week, while four schools (the control group) continued their normal activities with two physical education lessons per week. In addition to this, many children in both schools participated in externally organised sports activities, and the present article reports on this aspect of the CHAMPS-study DK.

### Participants and data collection

For the purposes of this study, we used the CHAMPS-study DK data collected between October 2008 and July 2011, meaning that we had data for boys and girls in school grades 0–4 in the first year of the study and grades 2–6 in the last year of the study. The data covered 2.5 years, minus the first 5 weeks of the study that were considered to be a pilot period for all participants. Data collection occurred weekly but was suspended during Christmas holidays and the 6 weeks of summer holidays. Since the CHAMPS-study DK was a natural experiment, entry to the study was kept open with the possibility for new children to enter and for others to exit if the family moved in or out of the school district.

#### Participation in sports

Organised leisure-time sport was assessed using weekly, automated text messages, SMS-track (https://sms-track.com/), with parents reporting how many times their child had participated in organised sport during the past week, using the relevant number between 0 and 7. If the child had participated more than seven times in such activities, the parent was asked to respond with ‘8’. One exposure unit per week would usually correspond to one hour of sport, based on knowledge of usual training time. In the event of a child participating in organised sport, a new question was automatically sent, asking for type of sport. Ten response options were offered (based on the most popular sports in the local community), with an instruction to type the relevant number(s) for the following sports: 1—soccer; 2—handball; 3—basketball; 4—volleyball; 5—rhythmic gymnastics; 6—tumbling gymnastics; 7—swimming; 8—horse-riding; 9—dancing; and 10—other sports.

Owing to the way in which these questions were set up in the SMS-track system, it was not possible to identify the number of exposures to separate sports, if children participated in more than one sport in a given week. For this reason, we restricted the data analysis to times (5-week periods) when only one specific type of sport was reported.

#### Musculoskeletal pain and classification of injury

Information on musculoskeletal pain was also collected weekly using SMS-track. Each Sunday, parents answered a text message asking about any musculoskeletal pain during the previous week: “Has (name of child) during the last week had any pain in: 1—neck, back, or low back; 2—shoulder, arm, or hand; 3—hip, leg, or foot; 4—no, my child has not had any pain”.

The returned answers were automatically recorded and inserted into a database. A reminder was sent if no response had been received within 72 hours and, if necessary, again 120 hours after the initial text message was sent. The SMS-track data were monitored and cleaned during data collection, and any inappropriate answers were checked through direct telephone contact with parents. If pain was reported by parents, a telephone consultation was carried out on the following Monday. If the pain had disappeared, the symptom was defined as ‘trivial’ and no further action was taken. If the pain had continued, it was considered ‘non-trivial’, and clinical examination was performed within the next fortnight by a physiotherapist, chiropractor or medical practitioner within the project team. If necessary, the child was referred for further paraclinical examination (such as X-ray, ultrasound or MRI) and possibly seen by a medical specialist. Information about any other treatment by an authorised health professional during the study period was also collected. Both the clinical and paraclinical examinations were free of charge.

The International Classification of Disease, 10th Revision (ICD-10) was used for diagnosis, and injuries were defined as either traumatic or overuse. Pain caused by an obvious injury (eg, fall, external knock) was diagnosed as a traumatic injury, whereas a painful condition was tentatively diagnosed as an overuse injury if (1) it was not caused by an obvious traumatic or sudden strain incident, (2) the painful area corresponded to a structure that had been used during a repetitive activity (such as a sport), and (3) this activity could be qualified as ‘overdoing’ relative to ordinary activities and for the constitution and/or fitness and strength of the child. The concept of overuse injuries has been extensively discussed in the literature and there appears to be no commonly accepted definition. However, we based ours on suggestions by Bahr.^15^ The present report deals exclusively with overuse injuries.

The primary outcome was the mean weekly incidence of overuse injuries in the upper and lower extremities. An incident case was defined as an OIE if it was preceded by at least one week without reported pain and was diagnosed as an overuse injury in the clinical examination.

### Ethical considerations

The Champs-study DK was approved by the regional ethics committee (ID S20080047) and was registered with the Danish Data Protection Agency. Schools were informed orally and in writing about the project, and written informed consent was obtained from the children's parents. Children and parents gave oral consent before any clinical examination. All participation was voluntary with the option to withdraw at any time.

### Statistical analysis

We first identified periods of 5 weeks in which children participated in only one type of sport (thus excluding 5-week periods with more than one sport). We did not report the number of sessions in which children participated in ‘other sports’, because with this category we were not able to distinguish if they practised one or more than one type of sport. Thereafter, we counted the number of sessions for each sport practised during these periods. For example, a child participating only in soccer for 25 weeks will be assigned to his/her first period from weeks 1 through 5, the second period from weeks 2 through 6, etc until the last period from weeks 21 through 25. Since these periods overlap, 21 periods will be registered for that child.

Incidence rates for injuries of the lower and upper extremities were determined by dividing the number of diagnosed overuse injuries by the total number of exposures for the specific sport. Weekly incidence rates (with 95% CI) were then calculated for each sport in relation to the number of weekly practice sessions.

A mixed effects logistic regression model was used to identify risk factors for injuries related to sport exposure reported during the 5-week periods. Covariates were selected from possible modifiers or confounders of the association between sport and injury: (1) sex, as boys and girls often participate in different sports, and boys may be less likely to be injured;^10^ (2) school grade (0–6) as a proxy for age (6–13 years), as it is unusual for Danish children to repeat a school year, and as different degrees of maturity are likely to affect choice of sport activity, risk and type of injury, and ability to report pain,^16^ and a positive link between age and risk of overuse injury to the extremities was shown previously in this study population;^11^ (3) school type (intervention or control school) because a difference in the number of weekly physical education lessons could affect the risk of an overuse injury and (4) school, class and individual ID as random variables to take account of any clustering effects.

Although previous injury has been shown to be predictor for future injuries,^17–20^ it was not included in our analysis, because a previous study from the same study sample showed this did not play a role in injuries of the extremities.^11^

Associations were reported as OR with 95% CIs. Since the study was explorative in nature, p values ≤0.05 were considered significant. All analyses were made using STATA 12.0.

## Results

A total of 1270 children (52% girls) participated during the study period, with 1218 children at baseline and 1226 at study completion (table 1). The maximum length of participation was 113 weeks, the average was 91.4 weeks, and the mean weekly response rate for the SMS messages was 96%. Study dropouts were mainly children moving away from the municipality, counterbalanced by new children moving to project schools.

**Table 1:** School grade, type of school and participation in sport for 1270 Danish schoolchildren included in the CHAMPS-study DK

	N	Boys (N=607) %	Girls (N=663) %
School grade at baseline			
0	227	47	53
1	253	47	53
2	272	54	46
3	252	45	55
4	266	45	55
Intervention school (6 weekly PE lessons)	741	45	55
Control school (2 weekly PE lessons)	529	51	49

Grade 0: 6–8 years; Grade 1: 7–9 years; Grade 2: 8–10 years; Grade 3: 9–11 years; Grade 4: 10–12 years.

PE, physical education.

Many children participated in more than one sport at a time, and were excluded from further analysis for technical reasons. During the 5-week periods when only one sport was reported, the most common activities were soccer, handball and swimming (table 2).

**Table 2:** Various leisure-time sport activities reported by 1270 Danish schoolchildren included in the Childhood Health, Activity, and Motor Performance School Study Denmark (CHAMPS-study DK)

Number of 5-week periods with only one sport	N
Soccer	15 490
Handball	8484
Swimming	5096
Horse-riding	4214
Tumbling gymnastics	3787
Rhythmic gymnastics	1789
Dance	1473
Volleyball	978
Basketball	905

Looking only at periods of single sport participation, overuse injuries of the lower extremities (n=292) were more common than injuries of the upper extremities (n=17) (table 3). To put this into context, during the whole 2.5 years of the CHAMPS Study-DK, there were 750 reported overuse injuries of the lower extremities and 40 overuse injuries of the upper extremities.

**Table 3:** Mean weekly incidence of overuse injuries of the extremities in relation to number of exposures per week during 5-week periods of single sport participation

	Lower extremity		Upper extremity	
Number of exposures to the sport in 5-week periods	Number of injuries N=292	Mean weekly incidence (95% CI)	Number of injuries N=17	Mean weekly incidence (95% CI)
Soccer (times)				
1–5	8	0.6 (0.2 to 1)	0	–
6–10	57	0.9 (0 to 1.1)	1	0 (0 to 0)
11–15	58	1.2 (0.9 to 1.5)	3	0.1 (0 to 0.1)
16–20	10	1.5 (0.6 to 2.4)	0	–
≥20	0	–	0	–
Handball (times)				
1–5	7	1.1 (0.3 to 1.9)	1	0.1 (0 to 0.4)
6–10	24	1 (0.6 to 1.4)	2	0.1 (0 to 0.2)
11–15	32	1.2 (0.8 to 1.6)	1	0 (0 to 0.1)
16–20	17	1.7 (0.9 to 2.5)	2	0.2 (0 to 0.4)
≥20	1	0.8 (0 to 2.5)	0	–
Swimming (times)				
1–5	10	0.4 (0.1 to 0.6)	1	0 (0 to 0.1)
6–10	2	0.2 (0 to 0.6)	0	–
11–15	1	0.9 (0.7 to 2.8)	0	–
16–20	1	1.3 (0. to 3.7)	0	–
≥20	0	–	0	–
Tumbling gymnastics (times)				
1–5	7	0.5 (0.1 to 0.9)	0	–
6–10	5	0.4 (0.1 to 0.9)	0	–
11–15	5	1.1 (0.1 to 2.2)	2	0.4 (0 to 1.1)
16–20	3	2.6 (0 to 5.6)	0	–
≥20	0	–	0	–
Horse-riding (times)				
1–5	6	0.4 (0.1 to 0.8)	2	0.1 (0 to 0.3)
6–10	8	1.3 (0.4 to 2.2)	0	–
11–15	4	0.7 (0 to 1.3)	0	–
16–20	2	0.5 (0 to 1.2)	0	–
≥20	3	0.4 (0 to 0.9)	1	0.1 (0 to 0.4)
Rhythmic gymnastics (times)				
1–5	2	0.3 (0 to 0.7)	1	0.1 (0 to 0.4)
6–10	1	0.2 (0 to 0.7)	0	–
11–15	0	–	0	–
16–20	0	–	0	–
≥20	0	–	0	–
Dance (times)				
1–5	4	0.6 (0 to 1.2)	0	–
6–10	2	0.5 (0 to 1.2)	0	–
11–15	0	–	0	–
16–20	0	–	0	–
≥20	0	–	0	–
Volleyball (times)				
1–5	0	–	0	–
6–10	3	0.8 (0 to 1.6)	0	–
≥11	0	–	0	–
Basketball (times)				
1–5	1	1.0 (0 to 3.2)	0	–
6–10	1	0.3 (0 to 0.9)	0	–
11–15	3	1.1 (0 to 2.4)	0	–
16–20	1	3.3 (0 to 9.9)	0	–
≥20	0	–	0	–
No sport at all	180*	0.5 (0.4 to 0.6)	9†	0.03 (0.02 to 0.06)
All population	750*	0.7 (0.1 to 1.2)	40†	0.04 (0.03 to 0.05)

*Not included in the total number of injuries (N=292) indicated at the top of the table.

†Not included in the total number of injuries (N=17) indicated at the top of the table.

Weekly incidence rates for overuse injuries to the lower extremities ranged from 0.2 and 3.3 for the individual sports, and rates for overuse injuries to the upper extremities were very low (table 3). There were no obvious dose–response patterns where the OIE rate increased with a higher level of sport participation.

The incidence estimate and during the 5-week periods was 0.69 (95% CI 0.65 to 0.74) for overuse injuries to the lower extremities and 0.04 (95% CI 0.03 to 0.05) for the upper extremities. As a comparison, children who were reported as not participating in any sport during 5-week periods had incidence estimates of overuse injuries of 0.49 (95% CI 0.41 to 0.56) for the lower extremities and 0.03 (95% CI 0.02 to 0.06) for the upper extremities.

Multivariate analyses showed that two sports (soccer and handball) were risk factors for overuse injuries to the lower extremities (table 4). Analyses could not be performed for several sports, or for overuse injuries to the upper extremities due to the small number of cases.

**Table 4:** Mixed effects logistic regression analysis of the mean weekly incidence of overuse injuries to the lower extremity by type of sport participation, controlling for gender, school grade and number of physical education classes (intervention/control school) with school, class and individual included as random variables

Number of sessions of each sport	OR (95% CI)	p Value
Soccer		
1–5	1.9 (1.4 to 2.6)	0.000
6–10	2.5 (1.8 to 3.4)	0.000
11–15	3.5 (2.4 to 5.0)	0.000
>15	4.2 (2.0 to 8.7)	0.000
Handball		
1–5	1.8 (1.3 to 2.9)	0.001
6–10	2.0 (1.46 to 2.39)	0.001
11–15	1.7 (1.1 to 2.8)	0.023
>15	1.52 (0.8 to 3.1)	0.241
Basketball		
>1	NA	NA
Volleyball		
>1	NA	NA
Rhythmic gymnastics		
>1	NA	NA
Tumbling gymnastics		
1–5	0.8 (0.5 to 1.3)	0.355
6–10	1.9 (1.0 to 3.3)	0.038
>10	NA	NA
Swimming		
>1	0.9 (0.6 to 1.2)	0.375
Dance		
>1	NA	NA
Horse-riding		
1–5	0.8 (0.5 to 1.4)	0.494
6–10	1.2 (0.5 to 3.0)	0.637
>10	0.8 (0.4 to 1.7)	0.590

NA, not available (because too few numbers in cells to perform analyses).

## Discussion

This study has confirmed that overuse injuries in children participating in sports are more common in the lower than in the upper extremities. Although the analysis was hampered by the low number of observations for some types of sports and for injuries to the upper extremities, we found that two sports—soccer and handball—were most strongly linked to overuse injuries of the lower extremities. The exposure-related incidence during 5-week periods was generally low, ranging from 0.2 to 3.3 per week. It is possible that overuse injuries are more frequent in the lower extremities due to the mechanical stress from the weight of the rest of the body, making the lower body more vulnerable than the upper extremities to extra strain from physical activity. Both soccer and handball put considerable stress on the body and, not surprisingly, children playing these sports were found to be most at risk of injury. Basketball is also likely to be a ‘risky’ sport, but the limited number of cases prevented analysis on this sport.

The results from, for example, two previous studies on the incidence of overuse injuries to the extremities in soccer are difficult to compare to those of this study, as their samples were from sports clubs (unlike ours from the general population) and incidence (per 1000 hours of the sport) was only reported for specific diagnoses, that is, lower extremity tendon pain and anterior leg pain/periostitis,^21^ and tendinopathy.^22^

Further, most studies, as in the previous two examples that were performed in sports clubs, use ‘time-loss’ as the criterion for injury. Clearly, the reporting of symptoms, the consultations of a clinician, or the inability to train and play (ie, time-loss) would result in completely different estimates of injuries. This study used a ‘hybrid’ definition, capturing children with potential injuries by text messages and actively contacting them to further explore the possible diagnoses. This would probably result in larger estimates than both ‘ordinary’ consultation figures and certainly larger than any time-loss estimate. This, in itself, makes comparison of incidence rates with most other studies irrelevant.

### Methodological considerations

Only 10 of the 19 schools in the municipality of Svendborg participated in this project, which could have resulted in selection bias. As far as we know, however, the participating schools were no different to the others, and it is likely that our results are generalisable to schoolchildren in other Danish municipalities. The results might have been different if we had been able to include injuries occurring during periods when children participated in more than one sport, but the method of data collection did not allow this.

For future studies, we recommend registering the number of hours of practice for each sport individually, which would make it easier to separate their effect on injuries and investigate the dose–response when children participate in several sports over the same time period. Injury information would have to specify also during which sport(s) symptoms have arisen. Moreover, we recommend reporting overuse injuries, both for the extremities and for the spine, in order to allow for comparisons which would bring a better overview of the whole musculoskeletal area.

Participation in the study was very satisfactory, and data collection via text messages seemed to be acceptable and relevant to parents as there were few missing data. Although 2.5 years is a long time for data collection, the risk of memory decay was minimised by the one-week recall period used throughout the study. A further strength was the conversion of self-reported injury data into ICD-10 diagnoses by specialised clinicians who met regularly to reach clinical consensus for the classifications.

Parents were chosen as informants rather than the children themselves, and this could be both a limitation and a strength. Parents will not know everything about their children's health, but self-report questionnaire data from young children may be inaccurate.^23^ Parental reports of their child's pain have previously been found to be reliable.^24^ SMS-track reporting in this study population was shown to have good validity when compared with verbal reporting with a specificity of 0.87, a sensitivity of 0.98 and a positive predictive value of 0.95.^25^

The incidence of sports injuries is typically investigated over a relatively long period (often one sports season) and reported as the number of injuries per 1000 hours of sport exposure. We chose to calculate the mean weekly incidence per hour of sport exposure in the previous 5 weeks, hoping that this would better identify overuse injuries due to repetitive exposure to the activity. Moreover, this strategy permitted investigation into the presence of dose–response.

## Conclusion

In this study population of schoolchildren who participated in organised sport activities outside of school, overuse injuries of the lower extremities were uncommon, and overuse injuries to the upper extremities were rare. Sports that combine weight-bearing with vigorous movements, such as soccer and handball, had the highest risk of overuse injuries. Our results suggest that overuse injuries in this population are not common, and that children can be encouraged to participate in organised sports activities outside of school.
